# Cells and signals of the leukemic microenvironment that support progression of T-cell acute lymphoblastic leukemia (T-ALL)

**DOI:** 10.1038/s12276-024-01335-7

**Published:** 2024-11-01

**Authors:** Aram Lyu, Seo Hee Nam, Ryan S. Humphrey, Terzah M. Horton, Lauren I. R. Ehrlich

**Affiliations:** 1 Division of Hematology/Oncology, Department of Medicine, University of California, San Francisco, CA USA; 2 Department of Molecular Biosciences, The University of Texas at Austin, Austin, TX USA; 3 Department of Pediatrics, Baylor College of Medicine/Dan L. Duncan Cancer Center and Texas Children’s Cancer Center, Houston, TX USA; 4 Department of Oncology, Livestrong Cancer Institutes, The University of Texas at Austin Dell Medical School, Austin, TX USA

**Keywords:** Cancer microenvironment, Acute lymphocytic leukaemia

## Abstract

Current intensified chemotherapy regimens have significantly increased survival rates for pediatric patients with T-cell acute lymphoblastic leukemia (T-ALL), but these treatments can result in serious adverse effects; furthermore, patients who are resistant to chemotherapy or who relapse have inferior outcomes, together highlighting the need for improved therapeutic strategies. Despite recent advances in stratifying T-ALL into molecular subtypes with distinct driver mutations, efforts to target the tumor-intrinsic genomic alterations critical for T-ALL progression have yet to translate into more effective and less toxic therapies. Ample evidence now indicates that extrinsic factors in the leukemic microenvironment are critical for T-ALL growth, infiltration, and therapeutic resistance. Considering the diversity of organs infiltrated by T-ALL cells and the unique cellular components of the microenvironment encountered at each site, it is likely that there are both shared features of tumor-supportive niches across multiple organs and site-specific features that are key to leukemia cell survival. Therefore, elucidating the distinct microenvironmental cues supporting T-ALL in different anatomic locations could reveal novel therapeutic targets to improve therapies. This review summarizes the current understanding of the intricate interplay between leukemia cells and the diverse cells they encounter within their tumor microenvironments (TMEs), as well as opportunities to therapeutically target the leukemic microenvironment.

## Targeting T-ALL microenvironments for better treatment outcomes

T-cell Acute lymphoblastic leukemia (T-ALL) is an aggressive, predominantly pediatric, cancer arising from excessive proliferation of transformed immature T cells in the thymus. Despite treatment advancements, patients experience significant side effects of chemotherapy, and those who fail to respond or relapse face poor outcomes, highlighting the need for more effective treatment strategies. This review examines how the tumor microenvironment supports growth and survival of T-ALL cells. The authors discuss current evidence indicating that interactions between T-ALL cells and the TME contribute to disease progression and treatment resistance in various organs, including the bone marrow, central nervous system, and spleen. These studies suggest that targeting interactions between T-ALL cells and the TME could offer new treatment opportunities. The authors conclude that disrupting the supportive signals of the TME could enhance existing therapies and reduce disease relapse.

This summary was initially drafted using artificial intelligence, then revised and fact-checked by the author.

## Introduction

Acute lymphoblastic leukemia (ALL) is one of the four primary types of leukemia, along with acute myeloid leukemia (AML), chronic lymphocytic leukemia (CLL), and chronic myeloid leukemia (CML). While AML, CLL, and CML primarily affect adults, approximately 80% of ALL cases occur in children^1,2^. ALL is classified into two main types: T-cell acute lymphoblastic leukemia (T-ALL) and B-cell acute lymphoblastic leukemia (B-ALL)^2,3^. T-ALL accounts for 15% of newly diagnosed cases in children and 25% in adults^4,5^. T-ALL circulates in peripheral blood^6^ and is characterized by the aggressive clonal proliferation of immature T-cell precursors originating in the thymus, which infiltrate secondary organs, including the spleen, the liver, bone marrow (BM), lymph nodes, and the central nervous system (CNS). Substantial progress has been made in T-ALL treatment in recent decades, leading to long-term remission in more than 90% of children and 60% of adult patients^7^. However, approximately 15–20% of pediatric and 50% of adult patients relapse or do not respond to initial therapy within two years of diagnosis^6^. Pediatric and adult patients with relapsed T-ALL have poor prognoses, with approximately 30–50% and 30% survival rates over 5 years, respectively^8^. Furthermore, current therapies cause adverse events, even in patients who are cured^9^. Therefore, there is an evident clinical need to develop less toxic therapies for T-ALL patients.

## Immunophenotypic and molecular characterization of T-ALL

T-ALL cells originate in the thymus as a deviation from canonical T-cell development. In leukemic blasts, oncogenes are frequently activated via chromosomal translocations between T-cell receptor (TCR) gene loci and coding and regulatory regions of genes encoding transcription factors^6^. T-ALL is classified into multiple subgroups on the basis of both phenotypic and genomic features. Initially, T-ALL subtype definitions were based on the immunophenotypes of leukemic blasts and their similarity to thymocytes at distinct stages of differentiation^10,11^. However, advances in sequencing technologies have revealed additional heterogeneity in the molecular subtypes of T-ALL due to coordinated dysregulation of gene expression programs, with a particular focus on classes of transcription factors. A recent genomic and transcriptional profiling study of T-ALL samples from a large pediatric cohort identified ten molecular subgroups, *HOXA*, *TLX3*, *TLX1*, *NKX2-1*, *LMO1/2*, *TAL1*, *TAL2*, *BCL11B*, *SPI1*, and T-other, which are distinguished by aberrant transcription factor expression, gene expression profiles, and genomic alterations^12^. This study highlighted several key biological pathways altered by genomic modifications in T-ALL, including transcription factor activity, epigenetic regulation, NOTCH signaling, cell cycle regulation, JAK-STAT signaling, and PI3K signaling. T-ALL subtypes are associated with distinct genomic alterations; for example, alterations in *SMARCA4*, encoding a transcriptional activator, are common in the *TLX3* T-ALL subtype, whereas alterations in the transcription factor *LEF1* are common in the NKX2-1 subtype. Alterations in *NOTCH1* and *CDKN2A* were prevalent in all T-ALL subtypes, occurring in 67% and 74% of T-ALL samples, respectively, relative to 0% and <30%, respectively, in B-ALL samples. Moreover, disruptions in genes related to RNA machinery account for 11% of T-ALL cases, with *NKX2-1*-rearranged T-ALL being driven by the RNA helicase *DDX3X*. Genomic alterations were also associated with differential patient outcomes in some subtypes; for example, in the TAL1 subtype, alterations in *PHF6*, encoding a transcriptional regulator, or in *PTEN*, a negative regulator of PI3K signaling, were associated with inferior event-free and overall survival. Below, we discuss correlations between major immunophenotypic and molecular features of T-ALL subtypes.

### Early T-lineage progenitor (ETP)-ALL

ETP-ALL, one of the three immunophenotypic subgroups of T-ALL, is associated with a high risk of relapse or failure to achieve remission, particularly in adults. ETP-ALL accounts for 10–15% of newly diagnosed pediatric T-ALL cases and results in a higher than typical rate of induction failure: ~6% for ETP-ALL versus ~1% for non-ETP-T-ALL^13^. Prior studies have identified ETP-ALL as a distinct subtype with notably poor responsiveness to chemotherapy in both pediatric and adult patients^14–16^. Compared with non-ETP-ALL patients, ETP-ALL patients have slower treatment responses, necessitating high-risk classification and more frequent consideration of hematopoietic stem cell transplantation^17^. Phenotypically, ETP-ALL blasts are characterized by flow cytometry with a CD1a^-^CD4^-^CD5^lo^CD8^-^ phenotype, indicating leukemic transformation of double-negative (DN; CD4^-^CD8^-^) immature thymocyte progenitors^14^. Dysregulated expression of the transcription factors LMO2/LYL1 and HOXA is common in ETP-ALL^18^. Moreover, ETP T-ALL exhibits distinct genomic characteristics and gene expression patterns compared to non-ETP T-ALL, often with fewer NOTCH1 mutations^18^, suggesting the involvement of alternative oncogenic pathways. In a recent subgroup analysis, ETP-ALL was most frequently associated with the HOXA and T-other molecular subgroups, which have inferior overall survival compared with most other subgroups^12^. Approximately 20% of pediatric ETP-ALLs have activating mutations in genes encoding interleukin-7 receptor (IL-7R) or the downstream Janus kinases JAK1 and JAK3, both of which are frequently altered in the HOXA molecular subgroup^12,19^. Consistent with an important role for aberrant IL-7R signaling in driving ETP-ALL, preclinical studies have shown that activating IL-7R mutations initiate ETP-ALL through blockade of thymocyte development^20^. ETP-ALL also aberrantly expresses multiple myeloid and hematopoietic stem cell markers, including CD13, CD33, and CD117, reflecting a progenitor state that precedes T-lineage commitment^14,19^. The similarity of ETP-ALL to immature progenitors is further supported by mutations in genes commonly altered in hematopoietic malignancies of other lineages, such as fms-related tyrosine kinase (FLT3) and isocitrate dehydrogenase 1 (IDH1), which are frequently mutated in myeloid leukemias^19^. Additionally, mutations in genes encoding transcription factors involved in hematopoiesis, such as runt-related transcription factor 1 (RUNX1) and ETS variant 6 (ETV 6), are characteristic of ETP-ALL^19^.

### Early cortical T-ALL

Early cortical T-ALL represents a subgroup with relatively favorable clinical outcomes^21^. This subtype accounts for 30–35% of pediatric T-ALL cases^22^ and is characterized phenotypically by CD1a^+^ membrane CD3 (mCD3)^-^CD4^+^CD8^+^ blasts, indicating a block at the early cortical double-positive (DP) stage of thymocyte maturation^21^. Common features of early cortical T-ALL include aberrant expression of the TLX1 (HOX11), TLX3, NKX2-1, and NKX2-2 transcription factors^18,23^. Moreover, this subgroup frequently exhibits gain-of-function mutations in NOTCH1 and loss of the tumor suppressor CDKN2A locus^18^. Early cortical T-ALL is strongly associated with elevated expression of genes involved in cell cycle progression (E2F7 and CDC2)^23^.

### Late cortical T-ALL

Late cortical T-ALL is the most prevalent subgroup of T-ALL, accounting for 35–60% of T-ALL pediatric cases, with blasts expressing a more mature cortical thymocyte immunophenotype (mCD3^+^CD4^+^CD8^+^)^22^. It is typically characterized by aberrant expression of TAL1 with either LMO1 or LMO2^18^. Late cortical T-ALL often presents with favorable patient outcomes^24^, with a particular trend toward improved event-free survival rates in patients with TAL1 rearrangements^25,26^. While late cortical T-ALL exhibits fewer activating mutations in NOTCH1, deletions of the CDKN2A locus are commonly observed in this subtype^18,27^. Moreover, the PI3K/AKT pathway is frequently activated through the loss of PTEN, a negative regulator of PI3K signaling, or through activating mutations in PI3K^18^.

## Current T-ALL therapies

Patients diagnosed with T-ALL typically undergo a 2-year course of risk-based multi-agent chemotherapy, with or without cranial radiotherapy (CRT)^28,29^. Treatment includes remission induction, consolidation, and maintenance therapy phases. The most significant predictor of patient outcomes is minimal residual disease (MRD) status at the conclusion of consolidation therapy. Risk stratification and treatment for adults and children with T-ALL vary. The Children’s Oncology Group (COG) has developed a risk classification schema for guiding pediatric T-ALL treatment, with ongoing efforts focused on optimizing conventional chemotherapy agents. While previous treatment regimens for T-ALL frequently included CRT to prevent T-ALL recurrence in the CNS, more recent therapies have shifted to intrathecal chemotherapy administration and/or higher doses of intravenous chemotherapy, leading to increased survival rates for pediatric T-ALL patients^1^. Moreover, the dose regimens of chemotherapy have evolved, with reduced anthracycline concentrations and increased utilization of asparaginase, dexamethasone, and high-dose methotrexate, and incorporation of nelarabine, particularly in high-risk groups^9,30^. Findings from the COG AALL0434 study, which was performed from 2009 to 2014 and involved 1256 children, adolescents, and young adults newly diagnosed with T-ALL, highlighted the significance of MRD monitoring for adjusting treatment intensity and improving outcomes^13^. The authors emphasized the importance of recognizing ETP-T-ALL patients for early risk assessment and appropriate clinical management. Patients with ETP-T-ALL, as well as near-ETP-T-ALL, exhibited slower responses to treatment than non-ETP-T-ALL patients, resulting in a higher rate of induction failure for the ETP-T-ALL and near-ETP-T-ALL subtypes. Participants with induction failure were treated with high-dose methotrexate and nelarabine. In the context of this more aggressive therapy, ETP-T-ALL and near-ETP-T-ALL patients did not experience inferior outcomes relative to non-ETP-T-ALL patients. Instead, MRD status after consolidation therapy best predicted overall outcomes irrespective of ETP subtype. In a more recent COG phase III trial for T-ALL (AALL1231), the treatment protocol employed dexamethasone and increased asparaginase, restricted CRT to the 10–15% of patients with CNS disease or persistent MRD positivity, and evaluated the potential benefits of incorporating bortezomib, an antineoplastic proteasome inhibitor, during induction and delayed intensification^31^. This study showed that limiting radiation to those with CNS3 disease or persistent MRD positivity was successful and decreased the incidence of long-term side effects from cranial radiation^13^ Together, current strategies use intensified chemotherapy regimens and optimized treatments, such as employing hematopoietic stem cell transplantation or CRT on the basis of MRD and CNS involvement, respectively, leading to significant improvements in overall treatment outcomes^13^.

## Rationale for investigating the tumor microenvironment (TME) in T-ALL

Despite the aforementioned therapeutic advances, survivors often face multiple morbidities, including secondary malignancies, cardiac issues such as decreased left ventricular ejection fraction, neurological complications, and endocrine disorders, significantly impacting their quality of life and contributing to premature mortality^32^. In addition to causing morbidity, current treatments can also result in selection of leukemic clones carrying mutations that confer chemoresistance, resulting in relapse^33,34^. Using xenograft models to evaluate clones present before and after chemotherapy, one study found genetic mutations in key human oncogenes and/or tumor suppressor genes, such as *PTEN* and *MYC*, resulting in elevated leukemia-initiating cell (LIC) activity in relapsed T-ALL^35^.

To identify novel targets for more effective treatment of primary and relapsed T-ALL, cell-intrinsic drivers of disease have been identified through genome-wide profiling approaches^12^, and ongoing clinical trials are evaluating the potential of targeting some of these factors^36^. Despite promising anti-leukemic effects observed in vitro and in preclinical studies, therapeutics targeting such cell-intrinsic drivers of disease have resulted in considerable systemic side effects. For example, gamma secretase inhibitors (GSIs) targeting hyperactivated NOTCH signaling lead to systemic toxicity in T-ALL patients, particularly affecting the gastrointestinal tract^37,38^. Similarly, drugs targeting the PI3K signaling pathway cause severe adverse effects, such as diarrhea and nausea, in patients with acute leukemias^39^. To date, the development of molecularly targeted therapies that significantly improve patient outcomes remains elusive. Thus, it is important to broaden our understanding beyond genetic and epigenetic changes to other factors that promote T-ALL progression and/or relapse. Despite multiple pro-leukemic genomic lesions, mouse and patient T-ALL cells are unable to survive well in vitro without supportive cytokines or signals from other components of the TME^40–43^, indicating that targeting the TME may be a promising novel therapeutic approach. To identify components of the TME that could be targeted to inhibit leukemia progression, it is important to consider the heterogeneity of cellular and molecular networks that leukemia cells encounter at the distinct sites they occupy, such as the BM and CNS. In this review, we discuss recent findings regarding contributions of multiple components of the TME to T-ALL pathogenesis. Leukemia-supportive signals provided by the TMEs of distinct organs will be discussed first before turning to perspectives on clinical strategies to target the TME.

## The role of tissue-specific TMEs in supporting T-ALL

### Thymus

**Fig. 1:**
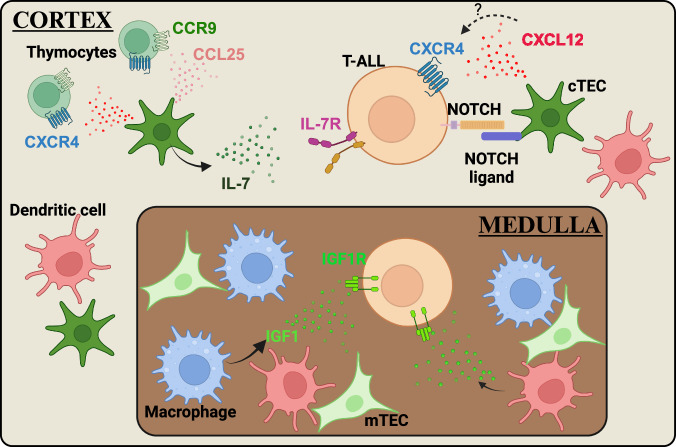
Mechanisms supporting T-ALL in the thymus microenvironment. The thymic TME promotes survival and proliferation of T-ALL cells by providing pro-leukemic signals such as IL-7, NOTCH ligands, and IGF1.

Thymocytes require bidirectional signaling with heterogeneous thymic stromal cells, including hematopoietic cells, such as dendritic cells (DCs), and non-hematopoietic cells, such as cortical thymic epithelial cells (cTECs), to develop properly and to support differentiation of the thymic stromal compartment^44,45^. Communication between thymocytes and TECs is critical for successful T-cell development. For example, cTECs express IL-7 and the NOTCH ligand DLL4, which are essential signaling molecules that promote survival, proliferation and T-lineage commitment of immature thymocyte subsets^46,47^. Given that aberrant activation of NOTCH and IL-7R signaling is common in T-ALL^5^, dysregulation of signaling pathways activated by cTECs could contribute to T-ALL initiation. Nascent leukemic cells in the thymus have the opportunity to exploit a wide variety of signals from the thymic microenvironment to support their own survival and growth. Therefore, understanding the signals between T-ALL cells and the cellular elements in the thymic TME could reveal potential targets for novel therapies (Fig. 1).

IL-7, a growth factor produced in the thymus primarily by cTECs and medullary thymic epithelial cells (mTECs), plays a key role in T-cell development^48–50^. IL-7 binds to a heterodimeric receptor composed of the IL-7Rα chain (CD127) and common cytokine-receptor γ-chain (CD132); ligand binding activates IL-7R-associated JAK1 and JAK3 tyrosine kinases^51^. IL-7R signaling is essential for survival and expansion of early T-cell precursors^52,53^ and facilitates survival of thymocytes following β-selection, a checkpoint that tests developing thymocytes for productive TCRβ chain gene rearrangements^54^. At this critical stage, thymocyte survival and proliferation are tightly controlled: cells that fail β-selection undergo apoptosis, while those that pass proliferate extensively, expanding the pool of progenitors with productive TCRβ rearrangements. IL-7R signaling is required to support proliferation of post β-selection thymocytes and prevent premature rearrangement of TCRα chain genes^55,56^. Given its essential role in thymocyte survival and proliferation, dysregulation of IL-7R signaling could promote leukemogenesis. In fact, overexpression of wild-type IL-7Rα induces T-ALL in mice, with activation of pathways including JAK/STAT, PI3K/AKT/mTOR, and NOTCH signaling, closely mimicking human T-ALL^57^. Furthermore, IL-7R is highly expressed by ~70% of adult and ~60% of pediatric T-ALL samples, regardless of IL-7R mutational status or T-ALL immunophenotypic classification, and this elevated expression of IL-7R renders T-ALL sensitive to JAK1 inhibition^58^. Together, these studies indicate that IL-7 in the leukemic TME could broadly promote progression of T-ALL. Consistent with this possibility, previous studies demonstrated that healthy thymic epithelial cells promote survival and proliferation of primary human T-ALL cells in vitro in an IL-7-dependent manner^59^. Furthermore, IL-7 produced by both bone marrow and thymic stromal cells supports survival and growth of primary patient T-ALL cells^60^. IL-7 also promotes leukemia expansion in mice engrafted with primary human T-ALL cells by downregulating the cyclin-dependent kinase inhibitor CDKN1B and upregulating the anti-apoptotic protein BCL-2^61^. Collectively, these findings implicate inhibition of IL-7 signaling as a promising thymic TME-based T-ALL therapy, which is in clinical evaluation, as discussed below.

NOTCH signaling is also essential for several stages of T-cell development, and activated NOTCH is present in the majority of T-ALL samples, where it acts as a critical oncogene to promote T-ALL leukemogenesis^62^. NOTCH signaling is required for immature thymocyte progenitors to commit to the T-cell lineage and for expression of transcription factors that regulate differentiation of CD4^-^CD8^-^ DN thymocyte subsets from early progenitors through β-selection^53^. While multiple NOTCH receptors (NOTCH1-4) are expressed by human thymocytes, NOTCH1 serves as the primary driver of T-cell development and commitment^63–65^. TECs express multiple NOTCH ligands, including DLL1, DLL4, JAG1 and JAG2, but only DLL4 is essential for T-cell development^46^. Upon ligand binding, NOTCH1 undergoes a series of proteolytic cleavages, first in the extracellular domain, initiated by an ADAM-family protease, and then at the transmembrane domain, mediated by the γ-secretase complex. These proteolytic events release the intracellular domain of the NOTCH receptor (ICN) from the membrane^66^, allowing it to traffic to the nucleus, where it promotes expression of target genes, such as *MYC* and *IGF1R*, that are critical for T-cell development, as well as for T-ALL initiation and progression^67–70^. Initial studies identified translocations between the TCR locus and the NOTCH1 gene that resulted in expression of a truncated, constitutively activated NOTCH1^71^. Subsequent studies revealed numerous additional mutations that activate NOTCH1 in T-ALL, resulting in both ligand-independent and ligand-dependent gain-of-function alleles, which are collectively present in the majority (>60%) of T-ALL patient samples^72^. *NOTCH1* mutations frequently occur in the heterodimerization domain (HD) and/or the C-terminal PEST domain^72^. Mutations in the HD region lead to ligand-independent activation of NOTCH1, whereas those in the PEST domain impair degradation of activated NOTCH1 but sustain the requirement for ligand binding to induce NOTCH signaling. In addition, mutations occur in genes regulating NOTCH1 activity, such as inactivating mutations in FBW7, an E3 ubiquitin ligase that promotes NICD degradation to terminate NOTCH signaling^73,74^. Although NOTCH1 functions as an oncogene critical for progression and LIC activity in T-ALL, primary patient samples with activating NOTCH1 mutations survive only when co-cultured with MS5 stromal cells expressing high levels of DLL1^40^, indicating a continued dependence on signals from cells in their microenvironment to activate NOTCH1 to support T-ALL cell survival. Therefore, targeting the components of the thymic TME that support activation of NOTCH1 signaling may be a promising therapeutic opportunity.

Insulin-like growth factor 1 receptor (IGF1R) represents a crucial downstream target of NOTCH signaling in T-ALL. IGF1R, a receptor tyrosine kinase, is essential for normal growth and development^75^ and is particularly important for early stages of T-cell differentiation during fetal development^76^. IGF1R binds to three ligands: IGF1, the primary high-affinity ligand, IGF2, and insulin^77^. While hepatocytes in the liver are the primary source of systemic IGF1^78,79^, small amounts are also produced by other cell types in multiple organs. This localized production of IGF1 has significant effects on the growth, survival, and differentiation of nearby cells^79^. Upon ligand binding to IGF1R, a series of downstream phosphorylation events activate both the PI3K–AKT and MAPK pathways. Dysregulated IGF1R signaling has been implicated in development and progression of various malignancies, notably including T-ALL^80,81^. NOTCH1 signaling results in elevated expression of IGF1R, and both pharmacologic and genetic perturbations of IGF1R signaling result in diminished T-ALL growth^70^. Additionally, T-ALL cells with reduced IGF1R expression exhibit diminished serial transplantation in mice, indicating reduced LIC activity^70^. Moreover, T-ALL cells expressing lower levels of IGF1R compensate by increasing PI3K-AKT activation, revealing the importance of this signaling pathway in T-ALL progression^70^. In line with these findings, in one study, pharmacologic inhibition of IGF1R decreased growth of a subset of human T-ALL cell lines, with sensitivity to IGF1R inhibition correlating with surface IGF1R expression levels and PTEN expression^82^. However, combined IGF1R and PI3Kγ inhibition did not effectively block growth of PTEN-negative T-ALL cells, suggesting the presence of additional mechanisms driving T-ALL progression^82^. IL-7 signaling played a distinct role in supporting T-ALL survival in this study, suggesting that its contribution is independent of the IGF1R-PI3K axis^82^. Local production of IGF1 can be critical for activating IGF1R signaling in T-ALL cells. Leukemia-associated myeloid cells, which directly support survival of mouse T-ALL cells in vitro^41–43,83–85^, produce IGF1. T-ALL cells show increased activation of IGF1R relative to healthy thymocytes, and IGF1R inhibition blocks myeloid-mediated T-ALL cell survival, demonstrating that other signals provided by myeloid cells do not override the requirement for IGF1R activation in vitro^41^. Subsequent studies revealed that tumor-associated myeloid cells promote initiation and progression of mouse T-ALL by activating IGF1R signaling in vivo^42^. Furthermore, human M-CSF-derived macrophages support primary patient T-ALL cells through IGF1R activation in cell culture^42^. Taken together, these findings suggest that blocking myeloid support could be a novel means of inhibiting IGF1R activation to specifically target survival of T-ALL cells.

### Bone marrow

**Fig. 2:**
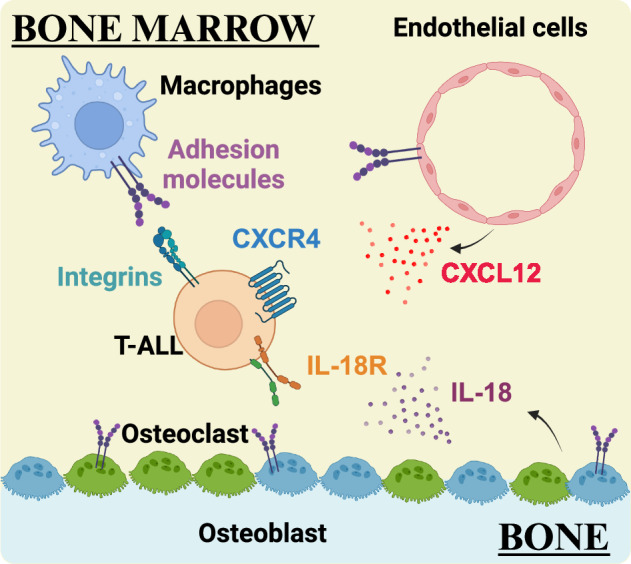
Signals within the bone marrow microenvironment that support T-ALL. The BM TME provides multiple signals including CXCL12, IL-18, and adhesion molecules, that support survival of T-ALL cells.

The bone marrow (BM) microenvironment plays a critical role in maintaining and regulating the differentiation of hematopoietic stem cells (HSCs) and supports leukemia progression^86^. Prior studies have highlighted the importance of signals within the BM niche in sustaining leukemic clones and promoting therapeutic resistance in various hematologic malignancies, including T-ALL^87,88^. Bidirectional interactions between T-ALL cells and diverse cellular components within the BM have been implicated in T-ALL pathogenesis; leukemia cells interact with both hematopoietic cells, such as macrophages and monocytes, and non-hematopoietic cells, such as endothelial cells, pericytes, osteolineage cells, and mesenchymal stem cells, in the BM TME^42,43,89–91^. Therefore, a deeper understanding of the interplay between leukemia cells and the diverse cellular components of the BM microenvironment holds promise for the identification of novel therapies for T-ALL patients (Fig. 2).

The BM serves as a crucial niche that provides factors that support HSCs^92^. Among these factors is CXCL12, also known as SDF1, a chemokine expressed by endothelial cells, osteoblasts, and mesenchymal stromal cells in the BM^93,94^. CXCL12 is the ligand for the chemokine receptor CXCR4, which regulates HSC maintenance. The CXCR4‒CXCL12 axis also supports T-ALL progression in a calcineurin-dependent manner in the BM^95,96^. Calcineurin (Cn) is a calcium-activated serine/threonine phosphatase critical for T-cell development^97,98^ that is also highly activated in T-ALL cells. Pharmacologic inhibition of Cn induces apoptosis of leukemia cells and prolongs survival in a mouse model of T-ALL^95^. Subsequent studies using a mouse model in which Cn deletion was restricted to T-ALL cells demonstrated that Cn was required for interactions between T-ALL cells and supportive stroma, as well as for leukemia progression and LIC activity^96^. Moreover, conditional deletion of Cn in T-ALL synergized with vincristine treatment, delaying T-ALL progression^96^. Additional studies revealed that Cn promotes expression of CXCR4 in T-ALL cells^90^ and that reduced CXCR4 expression in Cn-deficient T-ALL results in a migration defect in T-ALL cells that can be rescued by restoring CXCR4 expression^90^. Taken together, this study revealed that CXCR4 is essential for the motility, survival, and proliferation of mouse and human T-ALL cells, as well as their ability to home to the BM and support disease progression^90^. An independent study demonstrated that T-ALL cells closely interact in a CXCL12-dependent manner with a vascular endothelial cell niche in the BM^91^. CXCR4 is highly expressed on both mouse and human T-ALL cells, and genetic or pharmacologic perturbation of CXCR4 reduced T-ALL burden, prolonged mouse survival, and decreased LIC activity^91^. Collectively, these studies highlight a critical role for CXCR4 signaling in maintenance and progression of T-ALL in the BM TME, where the ligand CXCL12 is produced by endothelial cells. Given that T-ALL chemoresistance and recurrence in the BM correlate with inferior outcomes^99^, targeting the CXCR4‒CXCL12 axis in the BM TME represents a promising target for emergent T-ALL therapies.

The mitogen‐activated protein kinase (MAPK/MEK) signaling pathway is frequently activated in T‐ALL cells from adult patients^100^. Surprisingly, a previous study revealed that pharmacologic inhibition of MEK promotes growth and proliferation of patient T‐ALL cells when co‐cultured with stromal cells, while maintaining LIC activity^101^. This increased growth was found to be mediated by the secretion of IL‐18, a proinflammatory cytokine, by BM‐derived stromal cells following MEK inhibition^101^. IL-18 promotes activation and proliferation of T cells and T-ALL cells^102^. IL-18 was found to be elevated in the peripheral blood of both T‐ALL‐xenografted mice and T-ALL patients compared with controls, and IL-18 has been shown to support T-ALL cell survival both in vitro and in vivo^101^. These findings suggest that further investigation into the role of IL-18 in T-ALL pathogenesis is warranted.

The most common site of T-ALL relapse is the BM, where integrins play a key role in leukemia cell adhesion, migration and metastasis^103^. Integrins promote cell adhesion by binding a variety of ligands, including the extracellular matrix (ECM) components collagen, fibronectin, and laminin, which are present in multiple organs, including the BM^104,105^. Integrins also bind adhesion molecules, such as intercellular adhesion molecule 1 (ICAM-1) and vascular cell adhesion molecule 1 (VCAM-1), which are expressed by antigen-presenting cells (APCs) and endothelial cells to mediate cell‒cell contacts^104^. Integrin-mediated interactions of T-lineage cells with myeloid and stromal cells are essential for thymocyte development and selection and for T-cell migration, activation, and differentiation^44,106^. Given the cooperative signaling between integrins and growth factor receptors, including IGF1R^107^, and the fact that dysregulated integrin signaling promotes tumor growth and chemotherapeutic resistance^108^, integrin-mediated interactions may represent a prime therapeutic opportunity for T-ALL. Pharmacologic blockade of either the integrin lymphocyte function-associated antigen-1 (LFA-1) or its ligand ICAM-1 significantly diminished survival of human T-ALL cell lines co-cultured with BM stromal cells^109^. Moreover, BM stromal cells supported the survival of patient-derived T-ALL cells in co-cultures in an LFA-1:ICAM-1-dependent manner^109^. Integrin β1-mediated interactions between T-ALL cells and ECM components have also been implicated in the development of chemoresistance and relapse in T-ALL^110^. In one study, interactions between human T-ALL cells and collagen or Matrigel matrices enhanced T-ALL resistance to doxorubicin in an integrin β1-dependent manner; furthermore, blockade of integrin β1 reduced T-ALL burden in the BM, prolonged survival, and increased leukemia cell sensitivity to doxorubicin in a mouse model of T-ALL^110^. Chemotherapy resistance involved doxorubicin efflux via activation of the ABCC1 drug transporter and the focal adhesion kinase (FAK)-related proline-rich tyrosine kinase 2 (PYK2) pathway^110^. In addition to promoting interactions between T-ALL and stromal cells, integrins also play an important role in the interactions of T-ALL cells with myeloid cells in the BM of leukemic mice. T-ALL cells isolated from the BM of leukemic mice required leukemia-associated myeloid cells to survive in vitro^42^, and pharmacologic or genetic depletion of myeloid cells from leukemic mice led to a reduction in T-ALL burden across multiple organs, including the BM^42^. Subsequent studies used transcriptional profiling and in vitro transwell assays to demonstrate that integrin-mediated contacts between T-ALL cells and myeloid cells are required for T-ALL survival in vitro^43^. Blocking integrin ligands or inhibiting downstream FAK/PYK2 signaling not only reduced T-ALL burden across multiple organs, including the BM, but also prolonged survival of leukemic mice^43^. Additionally, inhibition of integrin-mediated adhesion or FAK/PYK2 signaling diminished survival of primary patient T-ALL cells co-cultured with peripheral blood mononuclear cell (PBMC)-derived myeloid cells^43^. Together, these findings demonstrate that integrin signaling, whether activated by interactions with the ECM, stromal cells, or myeloid cells, is a key pathway activated by the leukemic TME that promotes T-ALL survival. Thus, integrins and/or downstream signals could serve as promising therapeutic targets.

While the above studies suggest that multiple specific cellular interactions and signals in the BM TME support T-ALL progression, intravital microscopy revealed dynamic and promiscuous cellular interactions between T-ALL cells and multiple BM elements during disease progression that did not implicate any particular stromal element in supporting T-ALL^111^. Thus, further investigation into the interplay between T-ALL cells and diverse cellular components of the BM microenvironment is warranted.

### Spleen

**Fig. 3:**
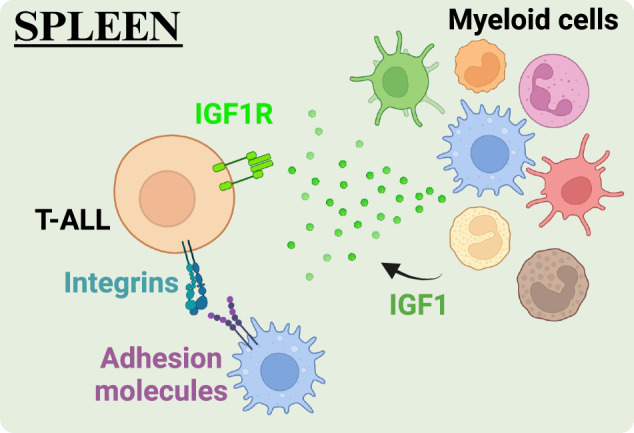
Mechanisms supporting T-ALL in the spleen. Myeloid cells play a key role in supporting T-ALL in the spleen by activating the IGF1R and integrin signaling pathways.

T-ALL cells are frequently detected in the spleen at the time of diagnosis^8^. Previous studies have highlighted the importance of extrathymic sites, including the spleen, in supporting T-cell development, particularly following BM transplantation^112,113^. These findings suggest that the splenic microenvironment may play a role in T-ALL initiation. A previous study revealed that surgical removal of the spleen prevented the development of T-ALL in a mouse model of DLL4-driven T-ALL^114^. Moreover, the spleen has been identified as a site of residual disease following chemotherapy in T-ALL patients^115^. Previous research emphasized the crucial role of the splenic environment in driving disease progression and inducing therapeutic resistance in multiple hematologic malignancies, including T-ALL^115–117^. CXCL12 is expressed by splenic stromal cells, including fibroblastic reticular cells^118,119^, potentially promoting T-ALL survival. Moreover, emerging evidence indicates that immune cells, notably myeloid cells in the splenic TME, play a role in driving T-ALL progression^41–43,83,85^. Therefore, exploring the cellular and molecular interactions between T-ALL cells and the splenic TME may aid in identification of therapeutic targets for T-ALL (Fig. 3).

The supportive function of myeloid cells in T-ALL relies on activation of IGF1R, which, as discussed above, is a critical growth factor receptor for LIC activity^70^. In multiple mouse models, T-ALL cells isolated from the spleen required myeloid support to survive in vitro^41^. Furthermore, pharmacologic or genetic ablation of myeloid cells led to a significant reduction in T-ALL burden across multiple organs, including the spleen and liver, thereby extending mouse survival^42^. Notably, IGF1R activation was reduced in splenic T-ALL cells following acute myeloid depletion in vivo, suggesting that IGF1R signaling is an important mechanism by which myeloid cells support T-ALL progression^42^. Together with the finding that enriched macrophage gene signatures are associated with inferior outcomes in pediatric T-ALL patients^42^, the potential of IGF1R signaling as a target for novel patient therapies is evident.

Despite the important role of the IGF1R pathway in myeloid-mediated T-ALL support, exogenous IGF1 is insufficient to sustain T-ALL cell survival in vitro, indicating a role for additional myeloid-derived signals in supporting leukemia cell survival. Notably, T-ALL cells rely on physical interactions with myeloid cells to survive in vitro^43^. Aberrant integrin signaling has been shown to drive disease progression and resistance to therapy across various hematologic malignancies, supporting leukemic cell survival and tissue invasion^108^. For example, integrin signaling enhances survival and proliferation of AML cells through the activation of the transcription factors STAT3 and STAT5, as well as the kinase Syk^120^. As discussed above, when mouse or human T-ALL cells interact with myeloid cells, integrin signaling activates FAK and PYK2^43^. Inhibiting ICAM-1 and VCAM-1, which respectively bind the integrins LFA-1 and VLA-4 that are expressed by T-ALL cells, or inhibiting FAK/PYK2 signaling reduced leukemia burden across multiple organs, including the spleen and liver, and prolonged mouse survival^43^. In PBMCs from pediatric T-ALL patients, elevated gene signatures of the integrin and FAK signaling pathways correlated with one another, as well as with increased myeloid gene signatures and unfavorable outcomes^43^. Taken together, these findings suggest that targeting integrin activation or downstream FAK/PYK2 signaling could be a therapeutic strategy for T-ALL in multiple organs, including the spleen.

### Central nervous system

CNS involvement occurs in approximately 10% of pediatric and adult ALL patients at diagnosis^121,122^, which is relatively more common than in other leukemias, such as AML, where the CNS is involved in approximately 1–2% of patients. Notably, the true incidence of CNS involvement may exceed reported rates because leukemia cell counts are below the level of detection at diagnosis in some patients^122,123^. Several studies have shown that infiltration of T-ALL cells into the CNS correlates with subsequent disease relapse and poor prognosis^124,125^. For example, in a study of pediatric ALL patients experiencing isolated CNS relapse, those who maintained an initial remission duration of over 18 months had a 4-year survival rate of approximately 78%, whereas individuals with a remission duration shorter than 18 months had a survival rate of approximately 51%^124^. Patients with the ETP-ALL subtype have been reported to be at an elevated risk of CNS involvement at diagnosis (e.g., in one study, 4 of the 6 ETP-ALL patients had CNS involvement)^15^.

**Fig. 4:**
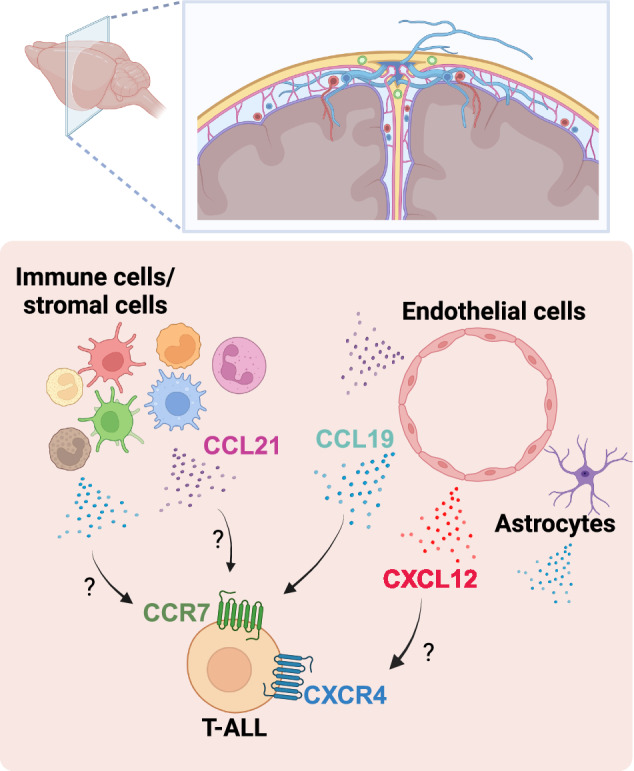
Mechanisms supporting T-ALL in the central nervous system. Multiples signals provided by the CNS TME, such as CCL19/21 and CXCL12, support entry and/or survival of T-ALL blasts.

Leukemic cell infiltration into the CNS can occur through multiple potential routes, including from the BM of the skull by bridging veins and into the cerebrospinal fluid via the choroid plexus^126^. Therefore, gaining a better understanding of the mechanisms supporting T-ALL trafficking to and survival within these regions, including the involvement of chemokines, is critical for developing efficacious treatment options, particularly for patients with CNS involvement who fail to respond to conventional therapies^127,128^. Here, we focus on molecular signals directly implicated in the infiltration of T-ALL cells into the CNS (Fig. 4).

CCR7 signaling has been identified as a critical signal for T-ALL trafficking to the CNS^129^. The CCR7 ligands CCL19 and CCL21 are produced in the CNS by diverse cell types, including macrophages, DCs, microglia, endothelial cells, and astrocytes^130–132^. In a NOTCH-induced mouse model of T-ALL, CCR7 expression by T-ALL cells was shown to be required for CNS infiltration, as was expression of CCR7 ligands by the leukemic host^129^. Furthermore, enforced CCR7 expression in human T-ALL cell lines was sufficient for T-ALL cells to enter the CNS^129^. Notably, CCR7 expression by T-ALL cells was induced by the activated NOTCH1 oncogene^129^. CARMA1, a signaling protein that plays a critical role in lymphocyte activation via the NFκB pathway^133^, is another key mediator of T-ALL infiltration into the CNS in mouse models^134^. Knockdown of CARMA1 in human T-ALL cell lines conferred a survival advantage to mouse hosts, and T-ALL patients with CNS involvement had elevated CARMA1 levels in the BM, supporting a role for CARMA1 in promoting CNS disease in T-ALL patients^134^. The diminished migration of T-ALL cells after knockdown of CARMA1 in response to CCL21 in vitro suggests that CARMA1 is linked to downstream signaling of CCR7, although CARMA1 and CCR7 could also independently regulate T-ALL migration and survival through additional signaling pathways^134^.

In addition to its established role in T-ALL initiation and progression within the BM, the CXCR4‒CXCL12 axis has also been implicated in the invasion of T-ALL cells into the CNS. Pharmacologic inhibition of CXCR4 significantly diminished the colonization of T-ALL cells in the BM and reduced neuropathologic aspects of the disease^126^. This study highlighted the important interplay between CXCR4-mediated BM colonization and infiltration of T-ALL cells into the CNS^126^. An additional study revealed that both ruxolitinib, a JAK1/2 inhibitor, and venetoclax, a BCL2 inhibitor, were ineffective in vivo for treating T-ALL in a mouse model because of leukemia cell infiltration into the CNS^135^. In this study, CXCR4 was found to be overexpressed in human T-ALL cell lines compared with healthy human T cells and was necessary for CNS infiltration^135^. Genetic deletion or pharmacologic inhibition of CXCR4 in T-ALL cells prolonged mouse survival and reduced T-ALL infiltration into the CNS, thus demonstrating the potential efficacy of targeting CXCR4 in combination with conventional chemotherapies^135^.

Additional recent evidence has highlighted the complexity of the CNS immune microenvironment, suggesting potential novel targets for increasing T-ALL patient survival rates. For example, the meninges have been shown to contain a diverse array of immune cells specific to CNS surveillance, including non-blood-derived monocyte and neutrophil populations originating in the cranial BM that populate the CNS borders^136^. Classical CNS macrophages, specifically meningeal and perivascular macrophages, undergo integrin-mediated interactions with vascular smooth muscle cells that are critical for their ontogeny and maturation. These studies demonstrate the potential for prominent and supportive interactions between myeloid cells and other cells in the CNS that have not yet been formally investigated in the context of leukemias^137^.

## Discussion and clinical perspectives

Although survival rates for T-ALL patients have significantly increased with current intensified chemotherapy regimens^29^, significant side effects, including neurotoxicity, seizures, and stroke-like symptoms, remain a major obstacle. As a result, research exploring therapeutic targets has broadened beyond identifying genetic alterations that impact the survival and progression of T-ALL cells, notably including elucidation of signals in the leukemia microenvironment. The significant role of the TME in promoting tumor survival and progression across various cancer types, including solid tumors and hematologic malignancies such as T-ALL, is widely recognized^88,138^. As discussed above, each leukemic organ comprises a unique TME with distinct supportive signals for T-ALL. Among the key components of the T-ALL TME, tumor-associated myeloid cells and associated signals have emerged as potential therapeutic targets, as they stand out for their presence across diverse anatomical sites. Myeloid cells promote T-ALL progression by activating IGF1R and integrin-mediated signaling in T-ALL cells^41–43^. Hypoxia also represents a potential therapeutic target in the TME; recent studies have shown that hypoxia in the BM inhibits T-ALL cell growth by slowing cell cycle progression and promoting resistance to antileukemic drugs^139^. Targeting hypoxia-induced factor 1α (HIF-1α), a key regulator of the cellular response to hypoxia, and activating the mTORC1 pathway may hold promise in overcoming drug resistance in T-ALL within the hypoxic BM TME^139^. Mesenchymal stem cells in the BM TME may also support T-ALL by accepting damaged mitochondria from leukemia cells through ICAM-1:integrin-mediated cell adhesion^140^. Overall, targeting signals provided by cellular components of the leukemic TME, along with inhibiting cell-intrinsic genetic alterations that drive T-ALL progression, has the potential to enhance the efficacy of T-ALL therapies.

Several clinical-stage inhibitors and antibodies that target signals activated by the TME have been assessed for their efficacy in treating T-ALL and other hematologic malignancies (Table 1). MK-0752, a NOTCH inhibitor, was evaluated in a phase 1 clinical trial for pediatric and adult T-ALL (NCT00100152). However, this study was terminated because of excessive toxicity related to on-target effects in the intestine, resulting in dose-limiting diarrhea^37^. CB-103, another pan-NOTCH inhibitor, was also evaluated in adult T-ALL patients (NCT03422679). Despite exhibiting favorable tolerability with fewer severe side effects, the clinical anti-tumor efficacy of this drug as monotherapy was limited^141^. BL-8040, a peptide-based CXCR4 antagonist, was evaluated in combination with chemotherapy in patients with T-ALL (NCT02763384). In this trial involving patients with relapsed or refractory T-ALL, BL-8040 was tested in combination with nelarabine. BL-8040 was well tolerated and resulted in complete remission in 5 of 9 adult patients^142^. These studies suggest that disruption of interactions between leukemic blasts and the BM TME could synergize with chemotherapy. A human monoclonal antibody against IGF1R, figitumumab (also known as CP-751,871), was studied in a phase I trial (NCT01536145) in multiple myeloma patients. Considering that this drug was well-tolerated, lowered granulocyte IGF1R expression, increased serum IGF1 levels, and reduced the tumor burden in 9 of 27 patients^143^, further exploration of its efficacy may hold promise for T-ALL patients. BI-505, a human anti-ICAM-1 monoclonal antibody, was evaluated in multiple myeloma patients (NCT01025206), and it demonstrated good tolerability, along with efficacy in 7 of 29 patients who had stable disease for at least 8 weeks. Given that BI-505’s efficacy was macrophage-dependent in preclinical studies, the authors suggest that it is most effective in patients with less impaired immune function and a lower tumor burden^144^. Recently, the IL-7Rα blocking antibody lusvertikimab (LUSV; formerly OSE-127) demonstrated suitable tolerability in a clinical trial involving healthy subjects, showing dose-dependent inhibition of IL-7 consumption without serious adverse events^145^. Preclinical studies have also shown that anti-IL-7Rα monoclonal antibodies, including LUSV, delayed T-ALL growth and prolonged mouse survival^146–148^, which inspired the current trials evaluating the inhibition of IL-7 signaling in T-ALL patients. Another approach to targeting IL-7R signaling employs the tyrosine kinase inhibitor ruxolitinib, which disrupts JAK 1 and 2 signaling downstream of IL-7R activation. Preclinical research and phase I and II clinical trials have assessed the safety and efficacy of ruxolitinib in treating various malignancies, including ALL (NCT02723994)^149^. In addition, trials investigating CCR7 inhibition in patients with hematologic malignancies are ongoing. Given promising results from preclinical studies on pro-tumor signals derived from the TME in T-ALL, further clinical trials targeting these pathways are warranted.

**Table 1:** Clinical trials evaluating the inhibition of tumor-supportive signals provided by the TME in various hematologic malignancies.

Target Signaling	Drug Name	Cancer type	Phase	Status	NCI identifier
NOTCH	MK0752 (γ-secretase inhibitor)	T-ALL	Phase 1	Terminated	NCT00100152
	CB-103 (pan-NOTCH inhibitor)	solid tumors, hematologic malignancies (NHL, T-ALL)	Phase 1 Phase 2	Terminated	NCT03422679
CXCR4-CXCL12	BL-8040 (CXCR4 antagonist)	T-ALL	Phase 2	Recruiting	NCT02763384
IGF1R	Figitumumab (anti-IGF1R antibody)	MM	Phase 1	Completed	NCT01536145
	XL228 (tyrosine kinase inhibitor against IGF1R)	CML, Ph+ ALL	Phase 1	Terminated	NCT00464113
Adhesion molecules	BI-505 (anti-ICAM-1 antibody)	MM	Phase 1	Completed	NCT01025206
		MM	Phase 2	Terminated	NCT01838369
IL-7	Lusvertikimab (anti-IL-7R antibody)	Healthy Subjects	Phase 1	Completed	NCT03980080
	Ruxolitinib (JAK1/2 inhibitor)	ALL	Phase 2	Active, not recruiting	NCT02723994
CCR7	JBH492 (anti-CCR7 antibody‒drug conjugate)	CLL, NHL	Phase 1	Active, not recruiting	NCT04240704
	CAP-100 (anti-CCR7 antibody)	CLL	Observational	Recruiting	NCT04704323

**Fig. 5:**
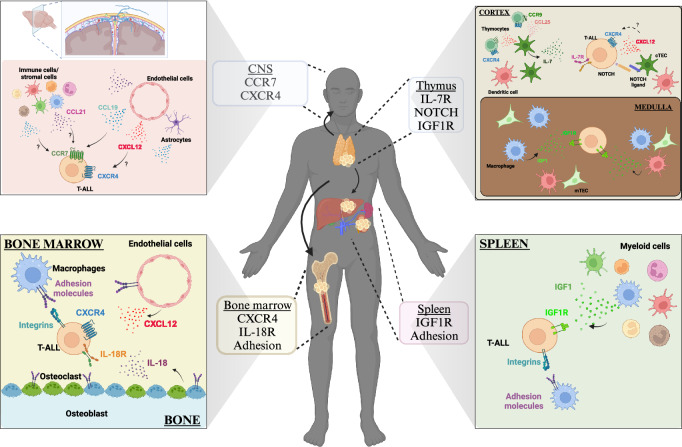
Summary of the diverse signals supporting T-ALL progression in the TME of multiple organs. Common and unique leukemia-supportive signals are provided by the the diverse TMEs where T-ALL invades, including of the thymus, BM, spleen, and CNS.

In conclusion, directing future T-ALL therapies against leukemia-supportive features of the TME introduces a novel opportunity, deviating from conventional genetic and epigenetic-centric approaches. Understanding the complex interplay between leukemia cells and cells in their surrounding microenvironment holds promise for developing more effective and less toxic treatments that improve patient outcomes while minimizing adverse effects. These recent discoveries carry substantial promise for refining the treatment of T-ALL, opening new opportunities for future therapeutic interventions (Fig. 5).
